# Effects of Hyporheic Water Fluxes and Sediment Grain Size on the Concentration and Diffusive Flux of Heavy Metals in the Streambed

**DOI:** 10.3390/ijerph14091020

**Published:** 2017-09-06

**Authors:** Qi Liu, Jinxi Song, Guotao Zhang, Weize Wang, Weiqiang Guo, Bin Tang, Feihe Kong, Aidi Huo

**Affiliations:** 1 Shaanxi Key Laboratory of Earth Surface System and Environmental Carrying Capacity, College of Urban and Environmental Sciences, Northwest University, Xi’an 710127, China; qiliu@stumail.nwu.edu.cn (Q.L.); wangweize@stumail.nwu.edu.cn (W.W.); weiqiangguo@stumail.nwu.edu.cn (W.G.); tangbin@stumail.nwu.edu.cn (B.T.); kongfeihe@hotmail.com (F.K.); 2 State Key Laboratory of Soil Erosion and Dryland Farming on the Loess Plateau, Institute of Soil and Water Conservation, Chinese Academy of Sciences and Ministry of Water Resources, Yangling 712100, China; 3 Key Laboratory of Mountain Hazards and Earth Surface Process, Institute of Mountain Hazards and Environment, Chinese Academy of Sciences, Chengdu 610041, China; zgt228@stumail.nwu.edu.cn; 4 University of Chinese Academy of Sciences, Beijing 100049, China; 5 School of Environmental Science and Engineering, Chang’an University, Xi’an 710054, China; huoaidi@163.com

**Keywords:** heavy metals, hyporheic vertical water exchange fluxes, sediment grain size, spatial variability, diffusive fluxes, Juehe River

## Abstract

The hyporheic zone regulates physicochemical processes in surface-groundwater systems and can be an important source of heavy metals in fluvial systems. This study assesses the pore water concentrations and diffusive fluxes of heavy metals with respect to the vertical water exchange flux (VWEF) and sediment grain size. Water and sediment samples were collected on August 2016 from upstream Site 1 and downstream Site 2 along the Juehe River in Shaanxi Province, China. Streambed vertical hydraulic conductivity ($K_{v}$) and the VWEF were estimated via the standpipe permeameter test method and Darcy’s law. The heavy metal concentrations in the pore water were measured and the diffusive fluxes were calculated using Fick’s first law. The VWEF patterns were dominated by upward flow, and Site 1 featured higher values of $K_{v}$ and VWEF. Higher Cu and Zn concentrations occurred near the channel centre with coarse sand and gravel and greater upward VWEFs because coarser sediment and greater upward VWEFs cause stronger metal desorption capacity. Additionally, Cu and Zn at the two sites generally diffused from pore water to surface water, potentially due to the upward VWEF. The VWEF and sediment grain size are likely crucial factors influencing the heavy metal concentrations and diffusive fluxes.

## 1. Introduction

The hyporheic zone (HZ) is defined as the saturated area beneath the riverbed where the mixing of groundwater and surface water generally occurs [1,2] and is also a dynamic area that acts as a source and sink of contaminants [3,4]. In streams and rivers, hyporheic exchange plays a significant role in nutrient and carbon dynamics and is an important factor in benthic habitats [5]. Hyporheic water exchange is an infiltration of stream water into the streambed and the re-emergence of this water into the stream after a certain period [6,7]. Shifts between upwelling and downwelling flow can intensely influence or change the transfer of nutrients and contaminants between the HZ and surface water or groundwater [8,9]. Additionally, hyporheic interstitial flows through the permeable sediment can easily transport dissolved oxygen (DO), nutrients, and dissolved metals into the HZ or stream, and these flows are probably affected by pressure gradients along the river bedforms, sediment grain size, and water discharge [10]. Hyporheic flow can further regulate and influence nutrient cycling and the transformation and diffusion of contaminants and heavy metals [4]. In general, the tracer-based methods can be used to estimate the hyporheic exchange, such as heat, environmental isotope, chloride and electrical conductivity [11]. The hyporheic vertical water exchange flux (VWEF) is one of the main factors influencing stream and groundwater quality [12]. The direction and magnitude of the VWEF through the HZ presents striking variability in space and time [13,14] and is closely related to the streambed vertical hydraulic conductivity ($K_{v}$) [15], sediment grain size [16], and streambed micro-topography [17]. Thus, it is important to understand the effects of hyporheic water exchange on the transfer processes of nutrients and contaminants. 

In recent years, water resources have continued to be seriously polluted by heavy metals related to mining and ore processing, and this pollution has become a severe problem of great concern throughout the world [18,19,20]. Trace heavy metals from different sources are introduced into a natural water body as part of the suspended load and as part of the sediment system due to absorption, precipitation, and ion exchange processes [21,22]. Heavy metals can be absorbed onto sediment particles, and the streambed sediment can easily release heavy metals into the pore water, potentially resulting in secondary pollution of the stream [23]. The sediment grain size can, to some degree, influence and control the transformation and diffusion of heavy metals in rivers, lakes, and oceans [24]. Heavy metal concentrations in sediment increased with decreasing sediment grain size [25]. Fine sediment particles have a strong absorption capacity for heavy metals, while coarse sediment particles have a strong desorption capacity for heavy metals, which means coarse sediments are more likely to release heavy metals into the pore water [26]. A significant negative correlation between fine sediment particles and streambed $K_{v}$/VWEF was reported by Wang et al. [13] for the Beiluo River in China, indicating that the VWEF in the HZ can influences the transport and diffusion of heavy metals [27]. Therefore, the heavy metal concentration in pore water is closely related to the sediment grain size as well as to the VWEF in the HZ.

For most heavy metals, the transport between streambed sediment and the pore water occurs via molecular diffusion [28,29], which represents the dominant transport mechanism at the sediment-water interface in marine, coastal, and lake systems [30]. Additionally, the occurrence of steep gradients in solute concentration between stream water and the HZ enhances the Fickian diffusion of solutes and metals, likely resulting in the higher diffusive fluxes at the sediment-surface interface [31]. Furthermore, the dissolved metals and nutrients can be driven by hyporheic pore water flow, which can transport material through permeable sediment. The transport rate of pore water is proportional to the permeability of the sediment and the pressure gradient [32]. However, the influences of sediment grain size and hyporheic VWEF on the pore water concentrations and diffusive fluxes of heavy metals remain unclear.

Thus, the main objectives of this study are to (1) determine the spatial variability in VWEF and streambed $K_{v}$ at the study sites; (2) investigate the spatial variability in heavy metal concentrations in pore water and diffusive fluxes at the sediment-water interface; and (3) further analyze the effects of VWEF and sediment grain size on the heavy metal concentrations and diffusive fluxes. 

## 2. Materials and Methods

### 2.1. Study Site

**Figure 1:**
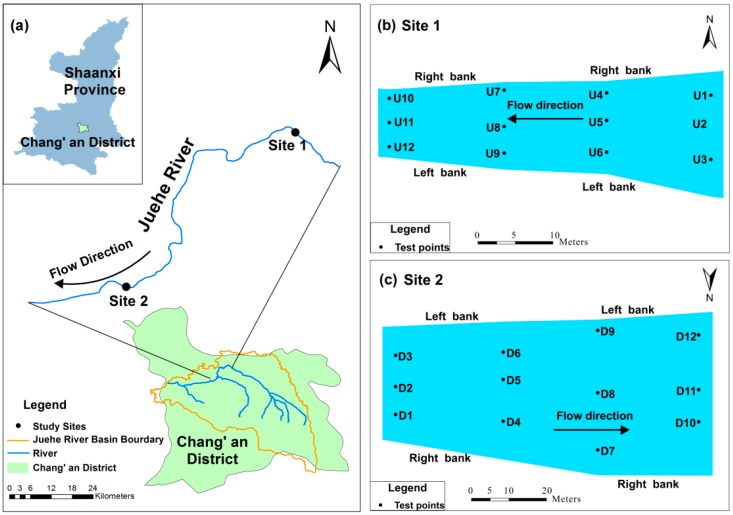
Map of the study sites and test points in the catchment showing: (**a**) location of the study sites within the Juehe River; (**b**) the measurement locations of the test points at Site 1 in the upstream, denoted by U1–U12; (**c**) the measurement locations of the test points at Site 2 in the downstream of Juehe River, denoted by D1–D12.

This field study was conducted in the Juehe River, which is a major tributary of the Fenghe River and a secondary tributary of the Weihe River, and was performed in the Chang’an District of Xi’an City (Shaanxi Province, China, Figure 1a). The Juehe River, with a total length of 64.2 km and a drainage area of 687 km^2^, originates in Dayiyu in the Qinling Mountains and merges with the Fenghe River at Qin Town of Xi’an City in Shaanxi Province. The river flows from east to west, and the elevation difference between the start and end of the river is approximately 54 m. In addition, it has an average stream gradient of approximately 2.83‰. The Juehe River Basin is part of the Weihe River Basin, characterized by a warm, temperate, continental monsoon climate with an annual mean temperature of 13.3 °C and a mean rainfall of 558–750 mm. The majority of the annual rainfall (78%) falls during the rainy season (from May to October) [33]. In contrast, very little natural runoff occurs in the dry season (from December to February).

During the campaign in 22–23 August 2016, two study sites in the Juehe River were chosen for measurements and sample collection, namely upstream Site 1 (34°09′02.28″ N, 108°54′56.05″ E) and downstream Site 2 (34°06′36.62″ N, 108°52′20.78″ E) (Figure 1a). At the two study sites, the left bank (relative to the stream flow direction) is an erosional bank, while the right bank is a depositional bank. A total of 24 test points were established along the right bank, centre and left bank of stream at the two sites. The mean stream flow rate was approximately 0.269 m/s, and river width ranged from 8 to 14 m from upstream to downstream. Each site contained four transects perpendicular to the flow direction, and each transect consisted of three test points (Figure 1b,c).

The materials in the streambed sediment along the flow direction varied. A sediment grain size analysis revealed that the streambed materials mainly consisted of deposited silt in the upper layer and fine sand in the lower layer at upstream Site 1 and consisted of 4 m of alluvial sand and gravel at downstream Site 2. The hydrological conditions and geomorphological features for each study site are described in Table 1.

### 2.2. Estimation of Streambed Vertical Hydraulic Conductivity ($K_{v}$) and Vertical Water Exchange Flux (VWEF)

**Figure 2:**
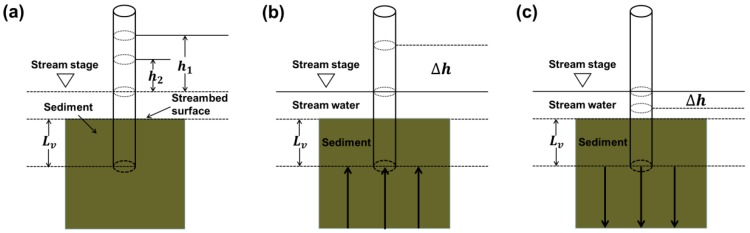
Schematic diagrams showing in situ permeameter tests for determination of (**a**) streambed vertical hydraulic conductivity ($K_{v}$) and vertical head gradient (VHG) for (**b**) upward flow and (**c**) downward flow.

In most previous studies, the streambed $K_{v}$ values were determined via the permeameter method [34,35,36,37]. In this study, falling-head permeameter tests were performed at 24 test points by inserting a vertical polyvinyl chloride pipe (PVC) (with an inner diameter of 5.4 cm and a length of 160 cm) into the streambed to a depth of approximately 0.45 m (Figure 2a). During the test, stream water was poured into the pipe to create a gradient in hydraulic head, and the water level of the pipe decreased. The duration of the falling head was recorded. Based on the hydraulic head and time data, the $K_{v}$ values were calculated using the solution of Hvorslev [38]:

(1) $$K_{v}=\frac{\frac{\pi D}{11m}+L_{v}}{t_{2}-t_{1}}ln\left(h_{1}/h_{2}\right)$$

where $L_{v}$ is the length of the streambed sediment column in the pipe; *D* is the interior diameter of the pipe; $h_{1}$ and $h_{2}$ are the hydraulic heads inside the pipe measured at times $t_{1}$ and $t_{2}$, respectively, and *m* is the ratio of horizontal hydraulic conductivity ($K_{h}$) and vertical hydraulic conductivity ($K_{v}$) of the streambed sediment (i.e., $m=\sqrt{K_{h}/K_{v}}$). When the length of the streambed sediment column ($L_{v}$) is five times larger than the diameter of the pipe (*D*), Equation (1) can be simplified as follows:

(2) $$K_{v}=\frac{L_{v}}{t_{2}-t_{1}}ln\left(h_{1}/h_{2}\right)$$

According to the research results reported by Chen [39], if the ratio of $L_{v}/D$ is commonly greater than five, the error of the modified calculation will be less than 5%. Therefore, Equation (2) can be used to calculate the $K_{v}$ values in this study sites.

The pipes were installed vertically in the streambed sediment and left for more than 16 h, and hydraulic head data were collected to determine the vertical head gradient (VHG). The VHGs at each test point were calculated using the method proposed by Freeze and Cherry [40]:

(3) $$\mathrm{VHG}=\frac{h_{i}-h_{j}}{L_{v}}$$

where $L_{v}$ is the length of sediment within the pipe, $h_{i}$ is the depth from the top of the pipe to the water level within in the pipe, and $h_{j}$ is the depth from the top of the pipe to the stream water level. 

A water level that is higher within the pipe than in the stream indicates an upward hydraulic gradient, reflecting inflow to the stream (Figure 2b). On the contrary, a water level that is lower within the pipe than in the stream indicates a downward hydraulic gradient, reflecting infiltration of stream water into the streambed (Figure 2c).

It is important to assume that the water exchange flux derived from Darcy’s law is vertical at the interface between the stream and streambed [41]. On the basis of the estimation of the streambed $K_{v}$ values and VHG, the VWEF at each point was calculated via Darcy’s law (Equation (4)) [35,42]:

(4) $$q_{v}=i\times K_{v}$$

where $q_{v}$ is the VWEF; i is the VHG; and $K_{v}$ is the vertical hydraulic conductivity.

### 2.3. Determination of the Heavy Metal Concentrations in Pore Water

A total of 72 sediment samples were collected from the two Juehe River sites after the permeameter test. In the sampling procedure, firstly, a PVC pipe was pressed vertically into the sediment to a measurement depth of approximately 0.45 m. Secondly, the top of a pipe was sealed using a rubber stopper after filling the pipe with stream water in order to separate the pipe from the atmosphere and prevent the release of the sediment [1,37], then the pipe was pulled out vertically. Thirdly, the sediment within the pipe was layered carefully, including 0–0.15 m, 0.15–0.30 m and 0.30–0.45 m. Finally, the sediment samples from different layers were packed into the sampling bags, labelled, and transported to the laboratory for analysis.

During the laboratory experiment in 24–26 August 2016, the collected streambed sediments from different layers were centrifuged at high speeds (5000 r·min^−1^ in 30 min) to extract the pore water. Approximately 30–40 mL of each pore water sample was collected and filtered through a 0.45 μm acetate cellulose filter membrane. Additionally, surface water and groundwater samples were also collected at each study site. The pore water samples were stored in pre-washed polystyrene bottles, and concentrated nitric acid (pH 2) was immediately added to bottles to preserve the samples until the heavy metal analysis. Prior to sampling, all sampling bottles were soaked in a 10% HNO_3_ solution for at least 24 h then thoroughly rinsed with de-ionized water to avoid contamination. All acidified pore water samples were stored at 4 °C and analysed within 48 h in the laboratory [43]. The concentrations of Cu and Zn in samples were determined by the Graphite Furnace Atomic Absorption Spectrophotometer (GF-AAS) (Z-2000, Hitachi, Tokyo, Japan). The detection limits of this method were 0.005 µg·L^−1^ for Cu and Zn, respectively. Three replicates of each sample were analysed for the heavy metal concentrations [44]. Therefore, the reported concentration result for each sample is the average value of the three replicates, thus reducing the relative standard deviations. 

### 2.4. Calculation of the Heavy Metal Diffusive Flux

Based on the concentration gradients in the pore water, the diffusive flux of the heavy metals was estimated from the pore water profile on the basis of the Fick’s first law [45]:

(5) $$F=\varphi \times D_{s}\times {\left(\frac{\partial c}{\partial x}\right)}_{x=0}$$

where F is the diffusive flux of heavy metal (µg·m^−2^·d^−1^), φ is the porosity of the surface sediment, *x* is the vertical depth beneath the sediment-water interface (cm), and $D_{s}$ is the molecular diffusion coefficient (cm^2^·s^−^^1^). ${\left(\frac{\partial c}{\partial x}\right)}_{x=0}$ is the concentration gradient of heavy metal across the sediment-water interface (µg·L^−1^·cm^−2^) and can be estimated from the porewater profiles, with the assumption that the gradient is one-dimensional [46] and can be approximated as [47]:

(6) $$\frac{dC}{dx}=\frac{C_{0}-C_{p}}{\Delta x}$$

where $C_{0}$ is the Cu or Zn concentration in pore water of the uppermost sediment layer (µg·L^−1^). $C_{p}$ is the Cu or Zn concentration in pore water (µg·L^−1^). ∆*x* is vertical depth difference beneath the sediment-water interface (cm).

The term $D_{s}$ is calculated using the equation of Ullman and Sandstrom [48]:

(7) $$D_{s}=\varphi D_{0}\;\left(\varphi \leq 0.7\right)$$

(8) $$D_{s}=\varphi^{2}D_{0}\;\left(\varphi >0.7\right)$$

where $D_{0}$ is the diffusion coefficient of ions at a given temperature 25 °C, $D_{0}$(Cu) = 7.33 × 10^−6^ cm^2^·s^−1^, $D_{0}$(Zn) = 7.15 × 10^−6^ cm^2^·s^−1^ [30]. Thus, positive values reflect a heavy metal diffusion direction from the sediment pore water to the overlying water, while negative values reflect the opposite trend.

### 2.5. Sediment Grain Size Analysis

All sediment samples were dried naturally and completely prior to the grain size analysis in the laboratory after pore water extraction. The dried sediment samples were homogenized and re-categorized using the sieving method through precleaned nylon screens. The streambed sediment classification based on Song et al. [49] suggests that silt and clay correspond to particle sizes of less than 0.075 mm, sand corresponds to particle sizes of 0.075–2.0 mm and gravel corresponds to particle sizes greater than 2.0 mm. Data from all size distributions were collected, and the cumulative percentage weights of sediment grain size and median grain size (*d*_50_ mm) were determined.

## 3. Results

### 3.1. Spatial Variability in Streambed $K_{v}$ and VWEF

The streambed $K_{v}$ values from 24 test points ranged from 0.005 to 88.501 m/d, with an average value of 25.614 m/d and a median value of 14.543 m/d. Hence, these values show a high degree of variability (Table 2). The VWEF values derived from the streambed $K_{v}$ and VHG also varied over a large range from −647.883 to 54.485 mm/d, with an overall average value of −178.741 mm/d for all test points (Table 2). The VWEF patterns at the 24 test points were mostly characterized by upward flow during the test period, which the exception of point U2 at Site 1 and points D1 and D7 at Site 2. 

**Figure 3:**
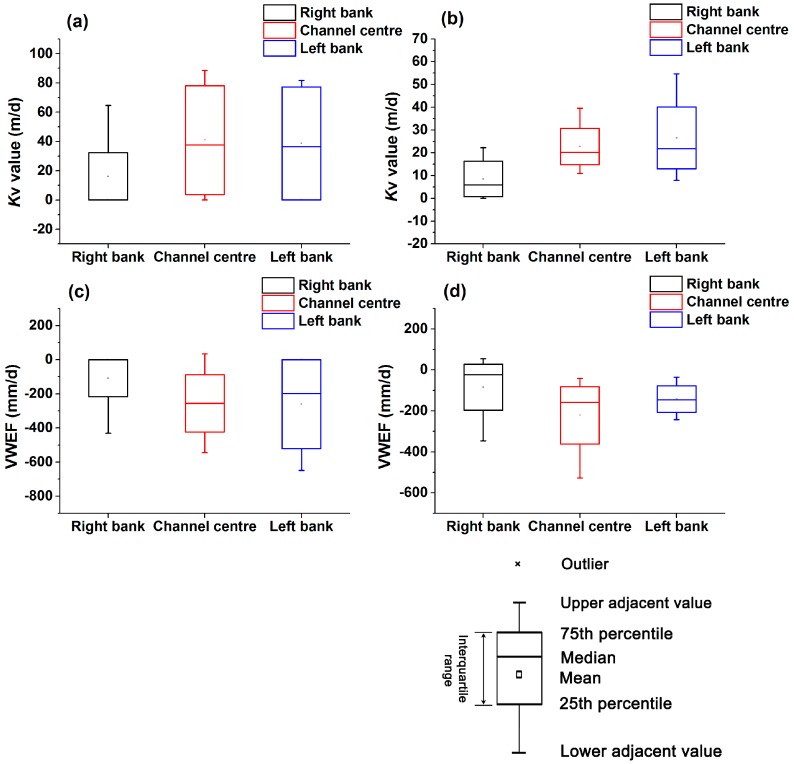
Box plots of spatial variation in streambed $K_{v}$ ((**a**) Site 1 and (**b**) Site 2) and VWEF ((**c**) Site 1 and (**d**) Site 2) in the Juehe River in August 2016 (Box indicates upper and lower quartile, the pane indicates the mean, the symbol line indicates the median, and the multiple is an outlier).

**Figure 4:**
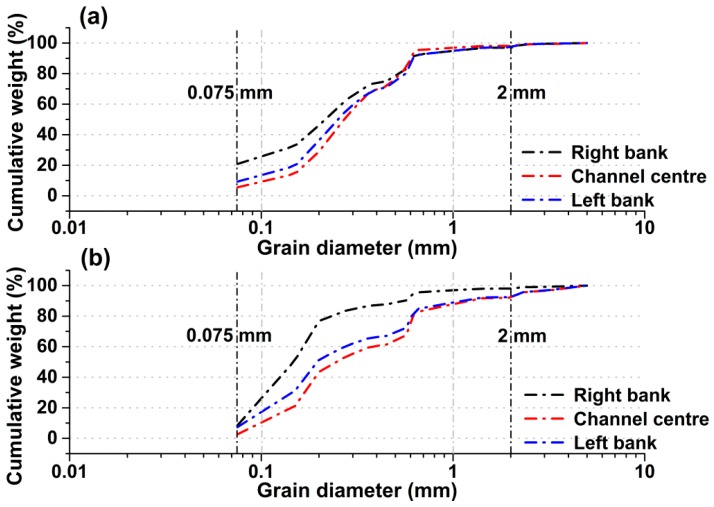
Average grain size distribution of streambed sediment at Site 1 (**a**) and Site 2 (**b**) along the Juehe River in August 2016.

However, the variabilities in $K_{v}$ and VWEF at Sites 1 and 2 during the test period were quantified (Figure 3). Upstream Site 1 featured wider ranges of $K_{v}$ values (0.005 to 88.501 m/d) and VWEF values (−647.883 to 34.410 mm/d) and higher average values of $K_{v}$ (31.936 m/d) and VWEF (−208.126 mm/d) than downstream Site 2 ($K_{v}$ = 19.293 m/d and VWEF = −149.357 mm/d) during the test period (Figure 3 and Table 2). In the channel cross-sections at Sites 1 and 2, lower $K_{v}$ values and upward VWEF were observed near the right bank, while higher $K_{v}$ values and upward VWEF occurred near the channel centre and toward the left bank of the stream (Figure 3). This pattern is likely related to the sediment grain size distribution, which affects the variability in pore space within the sediment [50]. As shown in Figure 4, the grain-size distribution varies significantly at different study sites. The streambed sediment at Site 1 mainly consisted of clay, silt, and coarser materials, while the sediment at Site 2 contained less clay and silt and a greater proportion of coarser materials (Table 3).

In general, the variability in $K_{v}$ values was always similar to the values of upward VWEF, indicating that the spatial variations in $K_{v}$ and VWEF were characterized by a “centre/left bank-high” and “right bank-low” pattern at Sites 1 and 2 [51]. Linear correlations between $K_{v}$ and upward VWEF based on the Spearman bivariate correlation analysis were obtained, and the correlation coefficients were 0.882 (*p* = 0.000) for Site 1 and 0.830 (*p* = 0.003) for Site 2. It further indicated a significant correlation between $K_{v}$ and upward VWEF in the two study sites. 

### 3.2. Spatial Variability in Cu and Zn Concentrations

**Figure 5:**
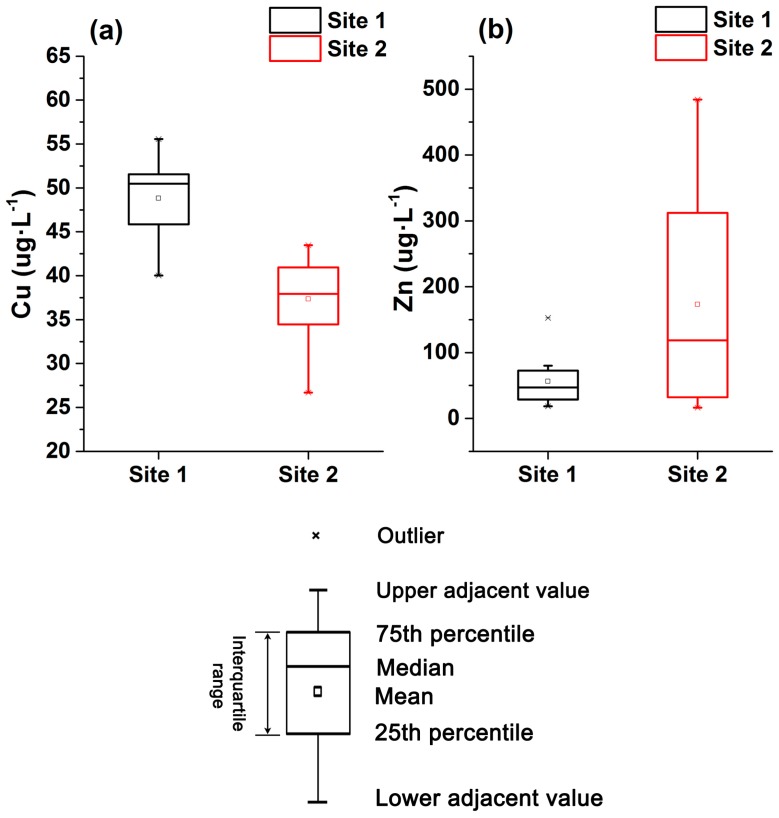
Box plots of spatial variations in Cu and Zn concentrations at the two study sites: Cu concentrations (**a**) and Zn concentrations (**b**) (Box indicates upper and lower quartile, the pane indicates the mean, the symbol line indicates the median, and the multiple is an outlier).

The average concentrations of Cu and Zn in the surface water, streambed pore water and groundwater at the two sites are shown in Table 4. At the 24 test points, the Cu and Zn concentrations were higher in the pore water (Cu = 43.15 µg·L^−1^, Zn = 124.31 µg·L^−1^) than in the surface water (Cu = 34.78 µg·L^−1^, Zn = 30.63 µg·L^−1^) and groundwater (Cu = 32.40 µg·L^−1^, Zn = 55.15 µg·L^−1^) (Table 4). The Cu concentrations ranged from 26.68 to 55.57 µg·L^−1^ in the pore water, from 26.7 to 38.1 µg·L^−1^ in the groundwater, and from 33.35 to 36.2 µg·L^−1^ in the surface water. The Zn concentrations ranged from 16.37 to 484.32 µg·L^−1^ in the pore water, from 25.25 to 36 µg·L^−1^ in the surface water, and from 34.65 to 75.65 µg·L^−1^ in the groundwater (Table 4). Therefore, pore water also exhibited larger variations in Cu and Zn concentrations during the test period. Additionally, significant differences in the pore water Cu and Zn concentrations were observed between Site 1 and Site 2 (Figure 5). The average concentration of Cu in the pore water was higher at Site 1 (48.81 µg·L^−1^) than at Site 2 (37.36 µg·L^−1^) (Figure 5a), whereas the average concentration of Zn at Site 2 (173.23 µg·L^−1^) was approximately three times greater than that at Site 1 (Figure 5b and Table 4).

**Figure 6:**
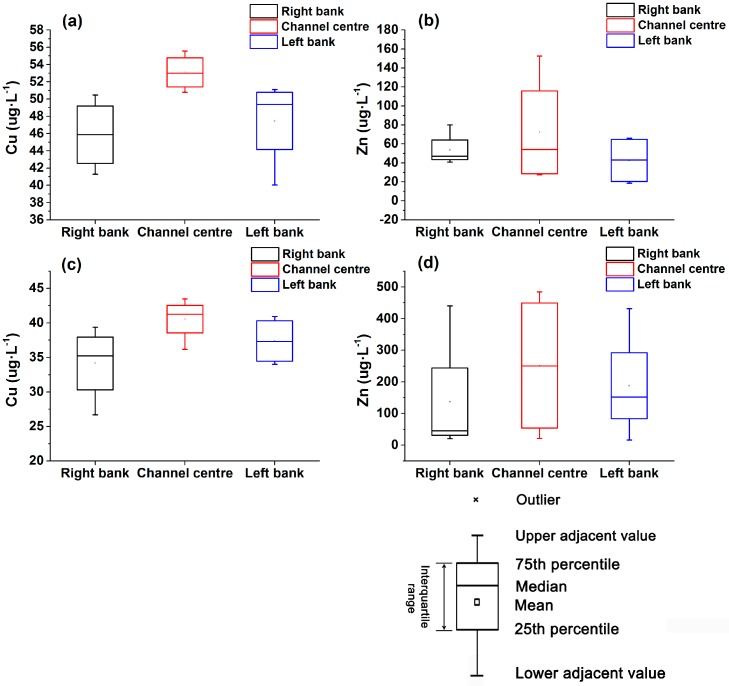
Box plots of lateral variability of Cu and Zn concentrations across the channel in the two sites: Site 1 (**a**,**b**) and Site 2 (**c**,**d**) (Box indicates upper and lower quartile, the pane indicates the mean, the symbol line indicates the median, and the multiple is an outlier).

**Figure 7:**
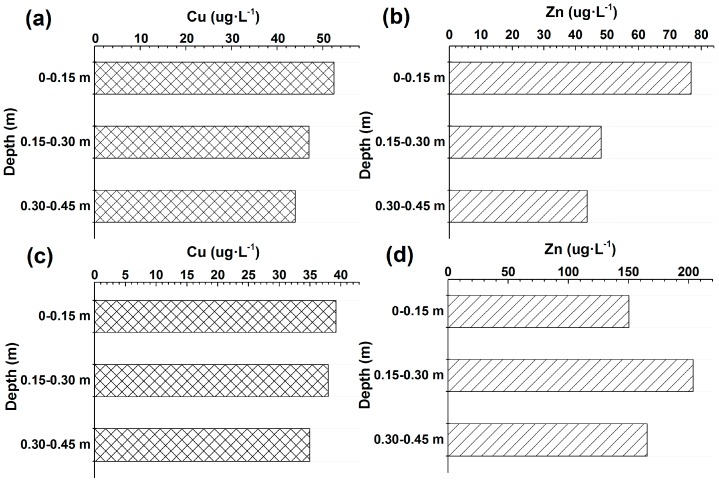
Vertical variation in Cu and Zn in pore water at different depths at Sites 1 and 2 ((**a**,**b**) indicate the Cu and Zn concentrations at Site 1, respectively; (**c**,**d**) indicate the Cu and Zn concentrations at Site 2, respectively).

In the channel cross-sections at Sites 1 and 2, higher Cu and Zn concentrations were observed near the centre of the channel, where higher upward VWEF always occurred (Figure 3c,d and Figure 6). Lower Cu and Zn concentrations were obtained near the right banks of the two sites, where lower upward VWEF generally occurred (Figure 3c,d and Figure 6). Therefore, the significant lateral spatial variability in Cu and Zn concentrations across the channel (including the centre, left bank and right bank) was always in accordance with the variation in VWEF at the study sites. Vertical variation in Cu and Zn concentrations in the sediment pore water are displayed in Figure 7. The Cu and Zn concentrations at the two sites were generally higher in the top layers (0–0.15 m) and decreased with increasing depth (Figure 7a–c). However, higher Zn concentrations in Site 2 were observed in the layer of 0.15–0.30 m (Figure 7d), thus creating an abrupt increasing trend from 0–0.15 m to 0.15–0.30 m and a decreasing trend from 0.15–0.30 m to 0.30–0.45 m.

### 3.3. Diffusive Fluxes of Cu and Zn in Sediment Pore Water

**Figure 8:**
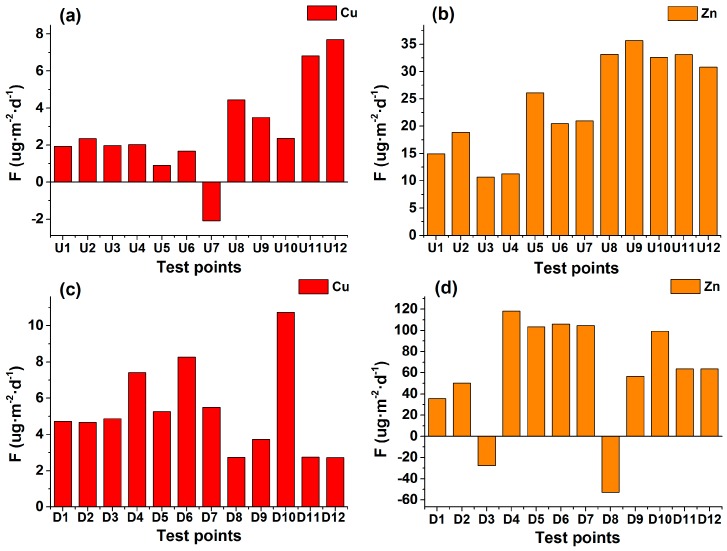
Diffusive fluxes of Cu and Zn at the sediment-water interface at Sites 1 and 2 ((**a**,**b**) indicate the Cu and Zn diffusive fluxes at Site 1, respectively; (**c**,**d**) indicate the Cu and Zn diffusive fluxes at Site 2, respectively).

The pathways of heavy metal transferring from the hyporheic zone include advection by upwelling ground water and Fickian diffusion [52]. Diffusion is the dominant transport mechanism across the sediment-water interface in marine, coastal, and lake systems [30]. The diffusive fluxes of Cu and Zn were estimated from the concentration gradients in pore water in order to determine the magnitude and pattern of heavy metal diffusion [45]. The diffusive fluxes of Cu and Zn in the pore water at the two sites were mostly positive (Figure 8), indicating the release of heavy metal contamination from pore water into the surface water. 

However, point U7 at Site 1 had a negative diffusive flux for Cu, while points D3 and D8 at Site 2 had negative diffusive fluxes for Zn (Figure 8a,d). Hence, at these points, the heavy metal contamination moves from the surface water into the pore water within the sediment. However, at the majority of test points, Cu and Zn were being released to the surface water from the sediment pore water. The release of these heavy metals may have negative effects on the surface water quality and river ecosystem health and may pose health risks and potential ecological risk [46]. Furthermore, higher positive diffusive fluxes of Zn (ranging from −53.014 to 118.187 µg·m^−2^·d^−1^) and Cu (ranging from 2.737 to 10.743 µg·m^−2^·d^−1^) were observed at the downstream Site 2 compared to the upstream Site 1, indicating significant regional differences (*p* = 0.033 for Zn and *p* = 0.025 for Cu) at the 95% confidence level based on the Two Independent Samples Test. Therefore, a Spearman bivariate correlation analysis was performed to determine the degree of correlation between upward VWEF and diffusive fluxes and to further analyze the influence of upward VWEF on the interfacial diffusion in the porous sediment layer. The resulting correlation coefficients between upward VWEF and diffusive fluxes of Cu and Zn were 0.455 (*p* = 0.038) and 0.469 (*p* = 0.032) for Site 1 and Site 2, respectively. Accordingly, significant positive correlations between the upward VWEF and diffusive fluxes of Cu and Zn in the two sites were observed during the test period.

## 4. Discussion

### 4.1. Spatial Variability in Streambed $K_{v}$, VWEF and Grain Size

In this study, the hyporheic VWEF patterns at the 24 test points at Sites 1 and 2 were mostly upward during the test period, which was subjected to the effects of groundwater discharging into the river at the gaining area. Other researchers have obtained similar results [4,53]. Spatial heterogeneity of $K_{v}$ might also influence the spatial distribution of groundwater recharge [54]. However, certain points, such as U2 at Site 1 and D1 and D7 at Site 2 presented local downward VWEF patterns, which were likely affected by the vertical temperature gradient at 45 cm depth and by micro-topography [55,56]. The heterogeneous distribution of the hyporheic VWEF through the streambed could be attributed to several factors, including streambed hydraulic conductivity [15], streambed topography [57] and heterogeneity of streambed sediments [8], contaminant discharges [58]. Additionally, there were significant positive correlations between $K_{v}$ and upward VWEF at the two sites, which is consistent with the results of Hyun et al. [15] and Binley et al. [2], suggesting that $K_{v}$ might represent a reliable estimation of VWEF. Hence, streambed $K_{v}$ can be a good indicator of the magnitude of VWEF at the test sites [59] and is likely driving factors that cause variations of VWEF in a fluvial system [13].

The spatial differences in $K_{v}$ and VWEF were obtained not only at the regional scale of the two sites (Sites 1 and 2) but also locally within each channel cross-section. The local variations were potentially affected by the dominant sediment grain size distributions at the study sites [60]. The streambed sediment materials, textures and grain size were critical controlling factors for streambed $K_{v}$ [61,62]. Hence, differences in the grain size distribution can lead to spatial variations in the $K_{v}$ [63], further influencing the distribution of hyporheic VWEF within each test site [13]. The streambed sediment at Site 1 mainly consisted of clay, silt, and coarser materials, while the sediment at Site 2 contained less clay and silt and a greater proportion of coarser materials (Table 3). These grain size differences led to a wider range of $K_{v}$ and VWEF values at Site 1. This result is similar to the research reported by Chen et al. [64], in which a $K_{v}$ difference of five orders of magnitude was obtained between silt/clay sediment and sand/gravel sediment. In the channel cross-sections, lateral variability in the grain size distributions among the left bank, centre, and right bank was observed at Sites 1 and 2 (Figure 4). The lowest cumulative percentage by weight of silt and clay and the highest *d*_50_ value occurred in the centre of the channel at Site 1 and along the left bank at Site 2. The highest cumulative percentage by weight of silt and clay was observed along the right bank of the channel at the two sites. The distributions of sediment grain size in the lateral direction at Sites 1 and 2 were to some degree related to the variability in the $K_{v}$ values and the VWEF during the test period. Most previous studies have found a significant negative correlation between the silt/clay content and the $K_{v}$ values [37,65] and significant positive correlations between *d*_50_ and $K_{v}$ and between *d*_50_ and VWEF [37,66]. Therefore, the Spearman correlation analysis was used to obtain the correlation coefficients between VWEF and $K_{v}$ (*r*^2^ = 0.906, *p* = 1.51472 × 10^−8^), silt/clay content (*r*^2^ = −0.596, *p* = 0.002), and *d*_50_ (*r*^2^ = 0.432, *p* = 0.035) for the two sites. The results were consistent with those of Wang et al. [13] for the Beiluo River in China. Thus, the grain-size distribution of streambed sediment is a critical factor that influences the spatial variability in streambed $K_{v}$ and VWEF and can, to some degree, be used to estimate the spatial variability in VWEF in fluvial systems.

### 4.2. Spatial Variability in Cu and Zn Concentrations in Pore Water and Their Diffusive Fluxes

The variability in the Cu and Zn concentrations in the pore water at the two sites was assessed. The Cu and Zn elements in the pore water showed relatively higher concentrations compared with the surface water and groundwater, which might result from the stronger desorption capacity of sediment particle or were driven by redox geochemistry, the co-precipitation or adsorption of metals with Fe or Mn oxyhydroxides [67]. Due to reductive dissolution, metals were prone to release into pore water. These heavy metal concentrations were closely associated with external pollution sources of heavy metals and other pollutants [43]. The highest Zn concentration occurred at Site 2, which is influenced by large amounts of domestic sewage and industrial wastewater that are discharged into the river between upstream Site 1 and downstream Site 2. The metals are likely transferred from upstream to downstream, leading to the accumulation of heavy metals within the sediment [43]. However, a higher Cu concentration was obtained at the upstream Site 1, which may be due to the higher Cu retention capacity of the sediment at Site 1 [68,69].

The spatial variability in heavy metal concentrations is mainly affected by grain size, hyporheic water exchange and anthropogenic factors [70]. Changes in heavy metals generally occur within the sediment-water system via adsorption, desorption, precipitation and ion exchange processes between solid and liquid phases [22]. The metal enrichment in the river is greatly dependent on the grain size distribution of the streambed sediment. Generally, the desorption capacity of heavy metal in coarse sand are higher than those in finer clay particles [21,71]. At Sites 1 and 2, the channel centre was predominantly composed of coarse sand (Table 3), which has a large heavy metal desorption capacity [26]. Hence, Cu and Zn were likely released from the coarse sand via desorption into the pore water in the centre of the channel, where the higher upward VWEF generally occurred (Figure 3). Furthermore, the heavy metal desorption capacity in the sediment-water system is enhanced by high upward VWEF conditions, thereby contributing to higher heavy metal concentrations in pore water (Figure 6). Thus, coarser sediment is associated with the higher upward VWEF, which can lead to increases in the sediment desorption capacity [24]. Accordingly, the mostly silt/clay composition of the sediment and the lower upward VWEF (Figure 3 and Figure 4) along the right bank of Sites 1 and 2 were associated with lower Cu and Zn concentrations.

Higher Cu and Zn concentrations occurred in the upper sediment layer, possibly due to organic matter degradation or oxidation [67,72]. These higher concentrations may be attributable to the accumulation of pollutants associated with surface runoff and human activities or a combination of shorter deposition time of surface sediment and high sedimentation rates leading to less organic matter oxidation at the sediment-water interface [73]. Cu is commonly bonded to organic matter via passive or active adsorption; thus, the burial of organic matter can lead to the transfer of Cu to the sediment surface [74].

The dissolved metal concentration gradient between the pore water and the surface water can not only reflect the transfer and diffusion directions of heavy metals but can also further influence the distribution and biological toxicity of heavy metals [75]. Studies have demonstrated that the transport of dissolved metals and nutrients is affected by hyporheic water exchange in fluvial system [27]. Pore water flow can transport dissolved material or contaminant through the permeable sediment into the river or from the river into the streambed sediment under the dynamic conditions of VWEF [32]. In this study, the hyporheic VWEF were dominated mostly by upward flow, which represented the average value on the whole at each point and promoted the release of heavy metals from the sediment pore water. However, there were individual points dominated by downward flow, which might be affected by benthos disturbances [1] and accelerated heavy metal precipitation into streambed. Therefore, the variability in the diffusive fluxes of Cu and Zn through the streambed at the two sites was related to the upward VWEF. Hence, VWEF is a good indicator of the diffusive fluxes of heavy metals through the streambed. Furthermore, in this study, the diffusive fluxes of Cu and Zn were primarily positive, indicating that the surface water was receiving heavy metal pollution from the HZ. This pollution could seriously affect the water quality of the river and generate potential ecological risks [76]. Thus, the HZ is an important pollution source of heavy metals, which is driven by the upward fluxes from the HZ into the stream. 

## 5. Summary

Thus, the findings obtained in this research can be summarized in the following three points:

(1) Vertical water exchange flux (VWEF) patterns in the two sites during the test period were dominantly upward flow and that higher value of $K_{v}$ and VWEF generally occurred at upstream Site 1, presenting a decreasing trend from the upstream to downstream.
(2) Higher Cu and Zn concentrations in pore water occurred near the channel centre with coarse sand and gravel and greater upward VWEFs because coarser sediment and greater upward VWEFs result in stronger heavy metal desorption capacity.
(3) Cu and Zn at the two sites generally diffused from sediment pore water to surface water, significant positive correlations between the upward VWEF and diffusive fluxes of Cu and Zn indicated that the diffusive fluxes were affected or controlled by the upward VWEF through the sediment.

## 6. Conclusions

In this study, the effects of hyporheic vertical water exchange fluxes (VWEF) and sediment grain size differences on the concentrations and diffusive fluxes of heavy metals through the streambed during the period of 22–23 August 2016 were analysed and discussed. The magnitudes and patterns of the VWEF at the study sites were estimated based on Darcy’s law using the streambed vertical hydraulic conductivity ($K_{v}$) and vertical hydraulic gradient (VHG). Additionally, the heavy metal concentrations in pore water were determined and the diffusive fluxes across the sediment-surface interface were calculated based on Fick’s first law.

The hyporheic VWEF patterns at the study sites were dominantly upwelling flow during the test period and the magnitudes of streambed $K_{v}$ and VWEF had a decreasing trend from upstream to downstream of stream. Meanwhile, the hyporheic VWEF and streambed $K_{v}$ exhibited spatial heterogeneity in a lateral cross-section. Higher values of $K_{v}$ and VWEF occurred in the centre of the channel and near the left erosional bank where the coarse sand and gravel gathered. Lower values of $K_{v}$ and VWEF appeared on the right depositional bank where the clay and silt gathered. Sediment grain-size distribution was a good indicator affecting streambed $K_{v}$ and VWEF.

Cu and Zn concentrations in pore water exhibited significant characteristics of spatial variation in different channel segments and a lateral cross-section. On the one hand, higher Cu concentrations occurred in upstream, while higher Zn concentrations occurred in downstream of channel. On the other hand, higher pore water concentrations of Cu and Zn were obtained in the vicinity of the channel centre, which was composed mostly of sand and gravel and featured high VWEF magnitudes, as well as were likely subjected to the comprehensive influences of greater metal desorption capacity of sand and gravel and driven by higher upward VWEF in fluvial system.

Additionally, the diffusive fluxes of Cu and Zn at the two study sites were mostly positive, indicating that heavy metals were transferred from the sediment pore water to the surface water. Significant positive correlations between the upward VWEF and diffusive fluxes of Cu and Zn were obtained for the study sites during the test period based on the Spearman bivariate correlation analysis, which indicated the diffusive fluxes were affected or controlled by the upward VWEF through the sediment. Furthermore, the hyporheic zone represents an important heavy metal pollution source and can cause secondary contamination, resulting in lower water quality in the river. 

We should undertake further research on the effects of hyporheic water exchange on the migration and transformation mechanism of heavy metal. The dynamical monitoring of heavy metals and hyporheic water exchange flux should be promoted to obtain the time-series data and further clarify the heavy metal migration with respect to hydrodynamics forcing in the hyporheic zone, which avoids the secondary contamination on the stream caused by the hyporheic zone and restores hydrological and ecological functions of river, and maintains the ecological balance.

## Figures and Tables

**Table 1:** The hydrological conditions and geomorphological features of the two study sites in the Juehe River in August 2016.

Study Sites	Site 1	Site 2
Test date	22 August 2016	23 August 2016
Coordinates	108°54′56.05″ E	108°52′20.78″ E
	34°09′02.28″ N	34°06′36.62″ N
Numbers of test points	12	12
Average width of stream (m)	7.825	14.38
Average velocity (m/s)	0.265	0.273
Max. water depth (cm)	30	61
Average water depth (cm)	23.50	41.75
Site description	Site 1 is a straight channel. Streambed sediment contains slit, clay, and coarser materials.	Site 2 is near the bend of stream. Streambed sediment contains medium sand, coarse sand and gravel.

**Table 2:** Statistical distributions of streambed $K_{v}$ values and VWEF for the two study sites.

Study Sites		Minimum	Maximum	Mean	Median	Std. Deviation (SD)	Coefficient of Variation (CV)
$K_{v}$ (m/d)	Site 1	0.005	88.501	31.936	3.789	36.995	1.158
	Site 2	0.011	54.674	19.293	18.377	14.957	0.775
	All	0.005	88.501	25.614	14.543	28.916	1.129
VWEF (mm/d)	Site 1	−647.883	34.410	−208.126	−106.005	236.990	−1.139
	Site 2	−527.107	54.485	−149.357	−121.292	156.370	−1.047
	All	−647.883	54.485	−178.741	−121.292	202.907	−1.135

**Table 3:** Sediment grain size distributions of Sites 1 and 2 in August 2016.

Grain Size		Site 1			Site 2		
		L	C	R	L	C	R
Average value of cumulative percentage (%)	0.075–2 mm (sand)	65.701	64.817	70.468	65.702	64.817	70.468
	<0.075 mm (silt-clay)	4.425	2.339	14.067	0.564	0.871	1.348
Average median grain size	$d_{50}$ (mm)	0.253	0.268	0.194	0.344	0.304	0.098

L, C and R represent the left bank, centre and right bank of the channel, respectively.

**Table 4:** Statistical distributions of Cu and Zn concentrations in surface water (SW), streambed pore water (PW), and groundwater (GW) collected at Site 1 and Site 2 in the Juehe River.

Study Sites			Minimum	Maximum	Mean	Median	Std. Deviation (SD)	Coefficient of Variation (CV)
Cu (µg·L^−1^)	Site 1	PW	40.03	55.57	48.81	50.48	4.63	0.09
	Site 2	PW	26.68	43.48	37.36	37.94	4.43	0.12
	All	SW	33.35	36.20	34.77	34.78	1.43	0.04
		PW	26.68	55.57	43.08	41.43	7.30	0.17
		GW	26.70	38.10	32.40	32.40	5.70	0.18
Zn (µg·L^−1^)	Site 1	PW	18.48	152.65	56.20	47.08	35.39	0.63
	Site 2	PW	16.37	484.32	173.23	118.70	167.14	0.96
	All	SW	42.40	130.20	86.30	86.30	43.90	0.51
		PW	16.37	484.32	114.72	55.83	134.23	1.17
		GW	36.30	108.90	72.60	72.60	36.30	0.50
